# SUCNR1 Is Expressed in Human Placenta and Mediates Angiogenesis: Significance in Gestational Diabetes

**DOI:** 10.3390/ijms222112048

**Published:** 2021-11-07

**Authors:** Reham Atallah, Juergen Gindlhuber, Wolfgang Platzer, Thomas Bärnthaler, Eva Tatzl, Wolfgang Toller, Jasmin Strutz, Sonja Rittchen, Petra Luschnig, Ruth Birner-Gruenberger, Christian Wadsack, Akos Heinemann

**Affiliations:** 1 Otto-Loewi Research Center for Vascular Biology, Immunology and Inflammation, Division of Pharmacology, Medical University of Graz, 8010 Graz, Austria; reham.atallah@medunigraz.at (R.A.); wolfgang.platzer@medunigraz.at (W.P.); thomas.baernthaler@medunigraz.at (T.B.); sonja.rittchen@medunigraz.at (S.R.); petra.luschnig@medunigraz.at (P.L.); 2 National Research Centre, Cairo 12622, Egypt; 3 Diagnostic and Research Institute of Pathology, Medical University of Graz, 8010 Graz, Austria; juergen.gindlhuber@medunigraz.at (J.G.); ruth.birner-gruenberger@tuwien.ac.at (R.B.-G.); 4 Institute of Chemical Technologies and Analytics, Technische Universität Wien, 1060 Vienna, Austria; 5 Department of Anaesthesiology and Intensive Care Medicine, Medical University of Graz, 8036 Graz, Austria; eva.tatzl@medunigraz.at (E.T.); wolfgang.toller@medunigraz.at (W.T.); 6 Department of Obstetrics and Gynecology, Medical University of Graz, 8036 Graz, Austria; jasmin.strutz@medunigraz.at (J.S.); christian.wadsack@medunigraz.at (C.W.); 7 Institute of Biomedical Science, Carinthia University of Applied Sciences, 9020 Klagenfurt, Austria

**Keywords:** succinate, SUCNR1, GDM, placenta, endothelial cells, angiogenesis

## Abstract

Placental hypervascularization has been reported in pregnancy-related pathologies such as gestational diabetes mellitus (GDM). Nevertheless, the underlying causes behind this abnormality are not well understood. In this study, we addressed the expression of SUCNR1 (cognate succinate receptor) in human placental endothelial cells and hypothesized that the succinate–SUCNR1 axis might play a role in the placental hypervascularization reported in GDM. We measured significantly higher succinate levels in placental tissue lysates from women with GDM relative to matched controls. In parallel, SUCNR1 protein expression was upregulated in GDM tissue lysates as well as in isolated diabetic fetoplacental arterial endothelial cells (FpECAds). A positive correlation of SUCNR1 and vascular endothelial growth factor (VEGF) protein levels in tissue lysates indicated a potential link between the succinate–SUCNR1 axis and placental angiogenesis. In our in vitro experiments, succinate prompted hallmarks of angiogenesis in human umbilical vein endothelial cells (HUVECs) such as proliferation, migration and spheroid sprouting. These results were further validated in fetoplacental arterial endothelial cells (FpECAs), where succinate induced endothelial tube formation. VEGF gene expression was increased in response to succinate in both HUVECs and FpECAs. Yet, knockdown of SUCNR1 in HUVECs led to suppression of VEGF gene expression and abrogated the migratory ability and wound healing in response to succinate. In conclusion, our data underline SUCNR1 as a promising metabolic target in human placenta and as a potential driver of enhanced placental angiogenesis in GDM.

## 1. Introduction

The placenta is a complex organ at the interface between the mother and the fetus. During the course of pregnancy, the placenta develops a large capillary network to allow for effective maternal–fetal exchange [1]. Hence, vasculogenesis and angiogenesis are crucial processes in the placenta and signs of villous network sprouting and vascular remodeling throughout gestation have been observed [2]. While regulation of placental angiogenesis by angiogenic factors in response to changes in oxygen tension and mechanical stimuli such as shear stress has been exhaustively addressed [1,3], an important aspect that has been largely overlooked is the possible contribution of placental metabolites to angiogenesis. In fact, the placenta has its own metabolic program which allows the tissue, on the one hand, to adapt to developmental changes and, on the other hand, to respond to stress. Thus, if this metabolic reprogramming fails, it can result in pregnancy complications [4].

GDM is defined as hyperglycemia first diagnosed during pregnancy and affects up to 18% of pregnancies [5]. Uncontrolled GDM can have long-term detrimental consequences for both the mother and the fetus, among which the risk of developing metabolic syndrome and Type 2 diabetes (T2D) is the most evident [6]. In GDM-complicated pregnancies, placentas exhibit augmented vascularization compared to normal pregnancies [7]. This can be explained in the context of fetal hypoxia arising from the upsurge in fetal oxygen demands in response to hyperglycemia and hyperinsulinemia [8]. Nevertheless, the exact mechanisms by which this placental anomaly occurs are not fully understood. 

A previous study showed that placentas from A2-GDM pregnancies (according to White’s classification, controlled by medication) showed compromised mitochondrial function relative to controls [9]. In addition, enhanced mitochondrial fusion in GDM placentas indicated deranged placental metabolism in GDM [10]. However, the alignment of the links between compromised placental metabolism and pregnancy complications is substantially unknown.

Succinate is a classically known intermediate metabolite in the Krebs cycle, [11] and its accumulation has been linked to conditions of insufficient oxygen supply [12]. Succinate has been shown to bind to the previously orphan G-protein coupled receptor GPR91 (now termed as SUCNR1) [13]. The signaling of succinate through SUCNR1 has been investigated in several organs, where it mediates a wide array of (patho-)physiological effects such as hepatic stellate cells activation in the liver [14], hematopoiesis in the bone marrow [15], inhibition of lipolysis in adipose tissue [16], regulation of ventricular cardiomyocyte viability in the heart [17] and renin release in the kidney [18]. It is now evident that succinate, through SUCNR1, provides a link between tissue metabolism, mitochondrial stress and organ response [19].

In the context of angiogenesis, Sapieha et al. showed that succinate induces angiogenesis through SUCNR1, both in normal retinal development and proliferative ischemic retinopathy [20]. Further, Mu et al. unraveled the role of succinate–SUCNR1 signaling in tumor angiogenesis [21]. Moreover, a link between succinate and synovial angiogenesis in rheumatoid arthritis was highlighted by Li et al. [22].

In this study, we set out to investigate the role of the succinate–SUCNR1 axis in human placental endothelial cells as major contributors and regulators of placental functions. We hypothesized that succinate–SUCNR1 signaling could play a role in placental angiogenesis, and might hence be suitable candidate molecules to tackle GDM-associated placental hypervascularization.

## 2. Results

### 2.1. Succinate, SUCNR1 Expression and VEGF Are Upregulated in Gestational Diabetic Placentas 

**Figure 1:**
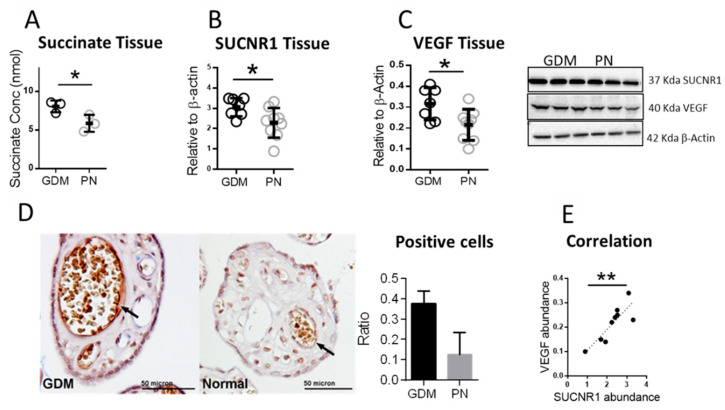
Succinate, SUCNR1 and VEGF upregulation in gestational diabetic placentas. (**A**) Succinate quantification in GDM and matched normal placental lysates using a spectrophotometric kit (*n* = 3). (**B**,**C**) Western blot of SUCNR1 and VEGF in whole tissue lysates from GDM (*n* = 7) and matched normal pregnancies (*n* = 9). (**D**) Immunohistochemistry staining of VEGF in placental tissue sections from GDM and normal pregnancy. Image is representative for 4 different placentas per group. Quantification of the images was performed with Fiji and the ratio of positive cells was calculated (*n* = 4). (**E**) Pearson correlation analysis of SUCNR1 and VEGF protein abundance in normal placental tissue (*n* = 9), ** *p* < 0.01, r = 0.85. Data in (**A**–**C**) were analyzed by unpaired *t*-test, * *p* < 0.05, data are shown as mean ± SD. Representative images and densitometry analysis are shown for (**B**,**C**).

Increased angiogenesis in the placenta is a key pathological feature of GDM [7]. However, our understanding of the mechanisms underlying this phenomenon is, as yet, incomplete. In this study, we aimed to investigate whether succinate and SUCNR1 are upregulated in GDM and whether there is an association between SUCNR1 expression and VEGF, as a key angiogenic factor, in the placenta. Thus, we used a colorimetric kit to measure succinate concentration in placental tissue lysates from GDM and matched normal placentas and found significantly elevated succinate levels in GDM placentas (Figure 1A). Additionally, we performed Western blot analysis for SUCNR1 in GDM and normal placental tissue lysates, where we observed significantly higher SUCNR1 protein abundance in GDM samples (Figure 1B). Similar results were obtained for VEGF in Western blot of placenta lysates (Figure 1C). We confirmed this finding by immunohistochemistry staining for VEGF in tissue sections that showed stronger staining intensity, particularly around the vessels, in GDM relative to normal sections (Figure 1D). A correlation analysis of the data for SUCNR1 and VEGF expression levels in tissue lysates shown in Figure 1E revealed a significant positive correlation. 

Taken together, these data revealed that succinate, SUCNR1 expression as well as VEGF were increased in GDM placental tissue lysates. In addition, SUCNR1 expression was correlated with VEGF abundance suggesting a potential role of SUCNR1 in placental angiogenesis through VEGF. 

### 2.2. Endothelial Cells in Human Placenta Express SUCNR1 

**Figure 2:**
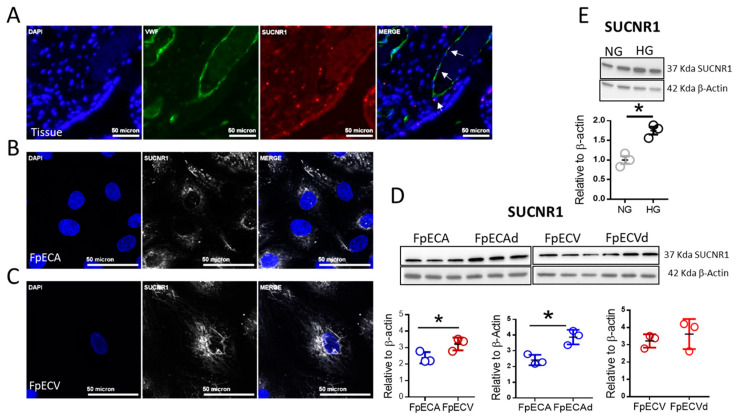
SUCNR1 expression in human full-term placental endothelial cells. (**A**) ISH combined with immunofluorescence staining of human full-term placenta. Tissue sections were stained with a probe against SUCNR1 and antibody against VWF as a marker for endothelial cells. Nuclei were counterstained with DAPI. Three sections from each placenta were examined and the shown image is representative for 5 normal placentas (*n* = 5). White arrows show co-expression of SUCNR1 and VWF. (**B**,**C**) Immunofluorescence staining of FpECAs and FpECVs, respectively, using antibody against SUCNR1 and DAPI for counterstaining. Images are representative for 3 different isolations (*n* = 3). (**D**) Western blot of SUCNR1 in isolated placental arterial and venous cells from GDM and normal pregnancies (*n* = 3). (**E**) Western blot of SUCNR1 in primary HUVECs cultured in normal or high glucose for 48 h (*n* = 3). For (**D**), unpaired *t*-test was used between groups, * *p* < 0.05, data are shown as mean ± SD (*n* = 3). For (**E**), paired *t*-test was performed, * *p* < 0.05, data are shown mean ±SEM. Representative images and densitometry analysis are shown for (**D**,**E**).

To further advance these observations, we performed a comprehensive analysis of SUCNR1 expression in human placenta. To this end, we combined In Situ Hybridization (ISH) of SUCNR1 mRNA with immunofluorescence staining of the endothelial cell marker von Willebrand factor (VWF) in human full-term placental tissue sections, and observed co-expression of SUCNR1 and VWF (Figure 2A). In addition, we performed immunofluorescence staining of isolated fetoplacental arterial and venous endothelial cells (FpECAs and FpECVs) using an antibody against SUCNR1, where we confirmed the receptor’s expression (Figure 2B,C). We then sought to compare the receptor expression in normal FpECAs and FpECVs versus diabetic FpECAd and FpECVd by Western blot. Interestingly, our data showed that in cells isolated from normal pregnancy, FpECVs expressed significantly higher levels of SUCNR1 relative to FpECAs. Moreover, we found that cells isolated from diabetic chorionic arteries expressed significantly higher receptor protein relative to controls, while this upregulation was less pronounced in venous cells (Figure 2D). Further Western blot analysis showed that HUVECs expressed similar SUCNR1 levels as FpECVs but comparatively lower levels than HEK293 cells and human monocytes derived macrophages as presented in Figure S1.

To test the hypothesis that hyperglycemia might account for the observed upregulation in receptor expression, we cultivated primary HUVECs, which we used for our in vitro experiments as endothelial cell model, in normal glucose (5.5 mM) as well as high glucose (20 mM). Indeed, we observed increased SUCNR1 expression in high glucose relative to normal glucose concentration by Western blot (Figure 2E). In summary, our data showed that placental endothelial cells express SUCNR1 and its expression in upregulated in diabetic isolations from chorionic arteries. Moreover, high glucose exposure of endothelial cells in vitro resulted in upregulation of SUCNR1 protein expression.

### 2.3. SUCNR1 Is Expressed on and within HUVECs 

**Figure 3:**
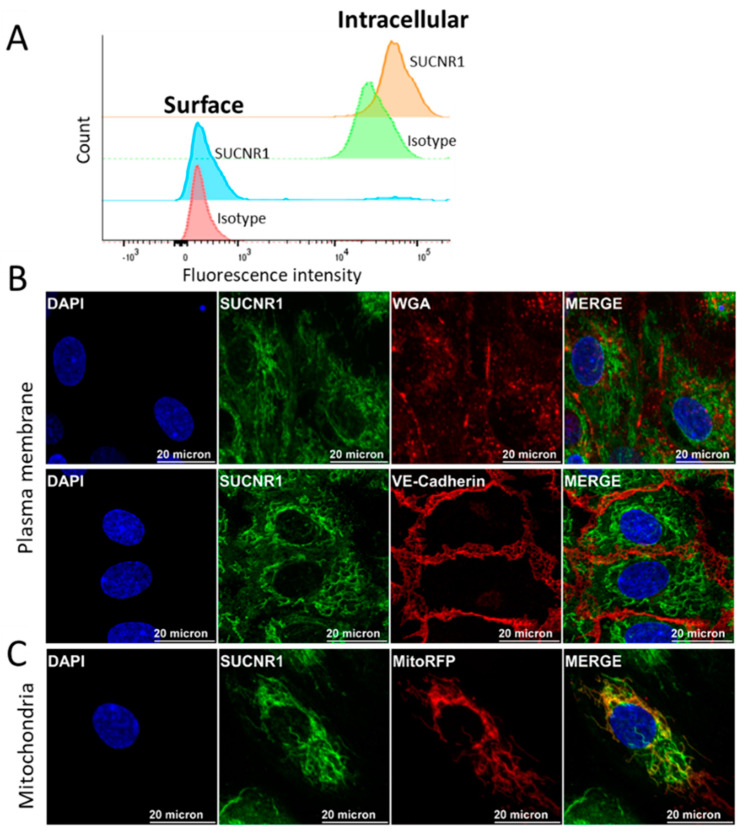
Surface and intracellular expression of SUCNR1 in HUVECs. (**A**) Flow cytometry staining of SUCNR1 in HUVECs with or without prior permeabilization steps (*n* = 3). (**B**) Immunofluorescence staining of SUCNR1 in non-permeabilized HUVECs labeled with plasma membrane markers (WGA: apical, and VE-Cadherin: basolateral sides) and DAPI. (**C**) Immunofluorescence staining of permeabilized HUVECs, using mitochondrial RFP, antibody against SUCNR1 and DAPI. For (**B**,**C**), images are representative for 5 different experiments (*n* = 5).

As the distribution of G protein-coupled receptors (GPCRs) between the plasma membrane and other intracellular compartments can impact their signaling, we attempted to address the localization of SUCNR1 in primary endothelial cells. Immunofluorescence staining of FpECs in Figure 2B,C was consistent with intracellular receptor localization. To investigate this further, we used primary HUVECs as model cells and performed flow cytometry after staining for SUCNR1 with or without permeabilization steps. We observed a shift in the signal with antibody staining relative to isotype control. This shift was more pronounced with permeabilization, indicating that the receptor was weakly expressed on the cell surface and prominently intracellularly (Figure 3A). To explore this further, we performed immunofluorescence staining of SUCNR1 in non-permeabilized HUVECs that were labeled with plasma membrane markers, WGA and VE-Cadherin. SUCNR1 signal was weak and partially colocalized with WGA but not VE-cadherin indicating that the surface receptor expression was limited to the apical but not basolateral side of the cells (Figure 3B). It was, indeed, interesting that most of the detected signal was derived from within the cells. Using mitochondrial RFP to identify the mitochondria in a subsequent staining of SUCNR1 in permeabilized cells unraveled clear colocalization (Figure 3C). In summary, SUCNR1 was found to be scantly expressed on the plasma membrane and mainly inside HUVECs, where it colocalized with mitochondria.

### 2.4. Succinate Induces Proliferation but not Apoptosis of HUVECs 

**Figure 4:**
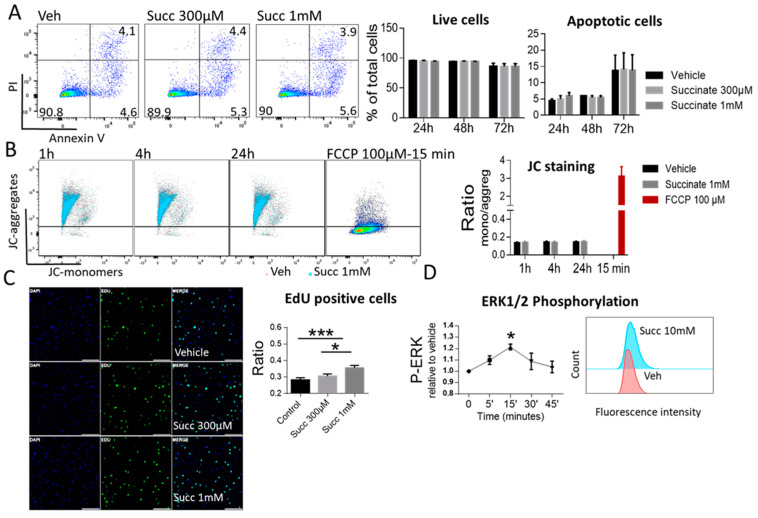
Succinate Induces proliferation but Not apoptosis of HUVECs. (**A**) Annexin V/PI staining of HUVECs treated either with vehicle or succinate. The percentages of apoptotic and live cells were calculated at 24, 48 and 72 h. (**B**) JC-1 staining of HUVECs treated with vehicle or succinate. FCCP, a mitochondrial membrane uncoupler, was used as a positive control. The ratio of JC monomers to aggregates was calculated from median fluorescence intensity at 1, 4 and 24 h. For A and B, representative flow cytometric dot plots are shown, data are mean ±SEM, (*n* = 3). (**C**) EdU proliferation assay of HUVECs treated with vehicle or succinate. At least 5 different fields per slide were examined and the ratio of EdU positive cells to the total number per field was calculated. Representative image is included (scale bar 200 µm). (**D**) ERK1/2 phosphorylation in EA.hy926 cells in response to succinate. Data are expressed as fold change in fluorescence intensity relative to vehicle. Representative histogram is shown. (**C**,**D**) were analyzed with one-way ANOVA followed by Tukey’s post hoc test, * *p* < 0.05, *** *p* < 0.001. Data are shown as mean ±SEM (*n* = 3-5).

To address the biological functions of the receptor, we investigated the response of HUVECs to the natural SUCNR1 ligand succinate. We performed annexin V/propidium iodide staining of HUVECs treated with vehicle or succinate for 24, 48 and 72 h. We evaluated the percentages of apoptotic and live cells and observed no differences with succinate treatment relative to vehicle at any time point (Figure 4A). In addition, we used JC-1 staining to assess whether the mitochondrial membrane potential was altered with succinate treatment. The ratio of JC monomers to aggregates at 1, 4 and 24 h revealed no alteration in succinate treated cells as compared to vehicle (Figure 4B). 

Next, we considered whether succinate might induce proliferation, rather than apoptosis, of HUVECs. Thus, we subjected HUVECs treated either with vehicle or succinate for 24 h to EdU proliferation assay. Indeed, we observed that succinate induced a concentration dependent increase in EdU positive ratio. This increase was significant with 1 mM succinate relative to vehicle (Figure 4C). Thus, our data confirmed that succinate did not affect endothelial cells viability, but rather promoted proliferation. In order to gain further insight into the mechanisms by which succinate induced this functional response in endothelial cells, and as SUCNR1 was reported to induce MAPK signaling cascade in numerous cell types [23], we used HUVECs cell line (EA.hy926) and examined EKR1/2 phosphorylation upon stimulation with 10 mM succinate. Our data showed that succinate in fact induced significant phosphorylation of ERK1/2 at 15 min after treatment (Figure 4D), while failed to induce AKT phosphorylation at any time point (Figure S2).

### 2.5. Succinate Enhances the Chemotactic Mobility, Wound Healing and Sprouting of HUVECs

**Figure 5:**
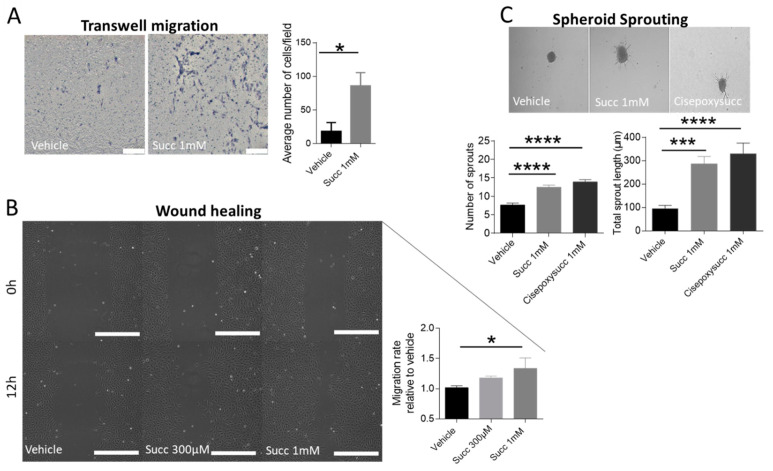
Succinate induces the chemotactic mobility, wound healing and sprouting of HUVECs. (**A**) Transwell migration assay of HUVECs in response to vehicle or succinate (scale bar 200 µm, objective 10x). Migrated cells were counted after 16 h on the lower surface of the filter and average numbers per field were calculated; paired *t*-test, * *p* < 0.05 (*n* = 4). (**B**) Scratch assay of HUVECs monolayer treated with vehicle or succinate. Distance migrated by cells over time was calculated and analyzed after 12 h. One-way ANOVA followed by Dunnett’s post hoc test was used, * *p* < 0.05 (*n* = 3, scale bar 500 µm, objective 10×). (**C**) Spheroid sprouting assay of HUVECs in response to succinate or *cis*-epoxysuccinic acid relative to vehicle indicated by total sprout length and number of sprouts. One-way ANOVA followed by Dunnett’s post hoc test was used, *** *p* < 0.001, **** *p* < 0.0001 (*n* = 5). For A, B and C, representative images are included and data are shown as mean ±SEM.

Keeping in mind that angiogenesis is a multi-step process that includes not only endothelial cell proliferation, but also migration and sprouting [24], we addressed whether succinate might act as a chemoattractant for HUVECs using a Transwell migration assay. The average number of HUVECs per field that had migrated towards succinate after 16 h was significantly higher than vehicle (Figure 5A). Next, we looked into the migration of the whole cell mass into wounded cell-free areas using a scratch assay. Time-lapse imaging revealed the ability of succinate to promote wound healing of HUVECs in a concentration-dependent manner, reaching statistical significance upon treatment with 1 mM succinate relative to vehicle (Figure 5B). Furthermore, we performed a 3D spheroid sprouting assay of HUVECs treated with succinate or vehicle. Analyzing cumulative (total) sprout length as well as sprout number showed significantly higher values for both parameters with succinate relative to vehicle (Figure 5C). We confirmed this result using a SUCNR1 agonist, *cis*-epoxysuccinic acid, identified by Geubelle et al. [25]. 

### 2.6. Succinate Boosts VEGF Gene Expression and Release in HUVECs 

**Figure 6:**
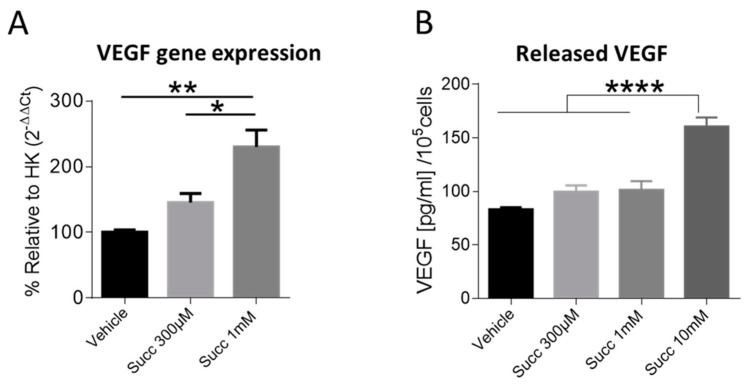
Succinate induces VEGF gene expression and release in HUVECs. (**A**) RT-PCR of HUVECs treated with vehicle or succinate for 4 h. (**B**) VEGF ELISA in culture supernatant of HUVECs treated with vehicle or succinate for 24 h. For A and B, one-way ANOVA followed by Tukey’s post hoc test was performed, * *p* < 0.05, ** *p* < 0.01, **** *p* < 0.0001, data are shown as mean ±SEM (*n* = 4–5).

So far, we showed that succinate promoted various angiogenesis correlates in HUVECs including proliferation, migration and sprouting. As VEGF is a principle mediator of physiological and pathological angiogenesis [26], we wondered whether succinate was able to increase VEGF gene expression. RT-PCR analysis in HUVECs treated with vehicle or succinate for 4 h, indeed, revealed a significant increase in VEGF gene expression in response to 1 mM succinate (Figure 6A). This was further corroborated by ELISA measurements at the protein level using conditioned media from HUVECs treated with succinate or vehicle for 24 h demonstrating that succinate caused an increase in VEGF secretion which reached significance with 10 mM succinate (Figure 6B).

### 2.7. Succinate Induces Capillary like Structures as well as VEGF Gene Expression in FpECAs

**Figure 7:**
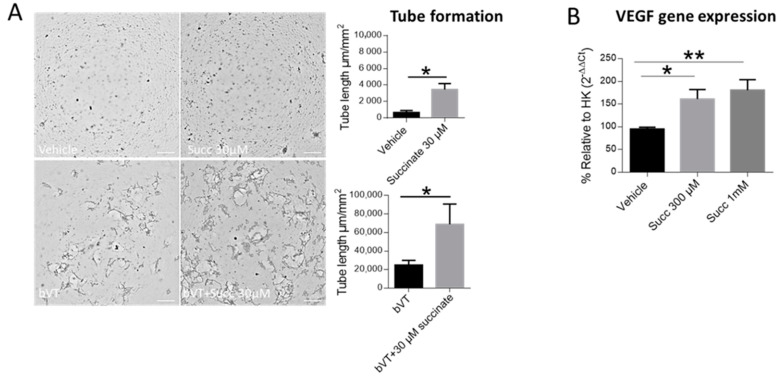
Succinate induces capillary structure formation as well as VEGF gene expression in FpECAs. (**A**) Tube formation assay in FpECAs treated with vehicle or succinate either alone or in combination with the positive control bVT. Tube length was calculated as µm per mm^2^; paired *t*-test, * *p* < 0.05, data are mean ±SEM (*n* = 4). Images are representative of 4 independent experiments (scale bar 500 µM, objective 4×). (**B**) RT-PCR of FpECAs treated with vehicle or succinate for 4 h and analyzed by one-way ANOVA followed by Dunnett’s post hoc test, * *p* < 0.05, ** *p* < 0.01, data are shown as mean ±SEM (*n* = 6).

Based on the aforementioned observations in HUVECs and being aware of the differences between venous and arterial cells [27], we investigated whether FpECAs showed an angiogenic response to succinate in a comparable manner as in HUVECs. With fibrin tube formation assays, we showed that FpECAs readily responded to low succinate concentration (30 µM). Tube length was significantly higher with succinate used alone or in combination with the positive control bVT (b-Fibroblast growth factor, VEGF and TNF-α) as depicted in Figure 7A. RT-PCR of FpECAs showed that like in HUVECs, treatment with succinate (300 µM and 1 mM) for 4 h significantly induced VEGF gene expression (Figure 7B).

### 2.8. Knockdown of SUCNR1 Reverses the Angiogenic Phenotype of HUVECs in Response to Succinate 

**Figure 8:**
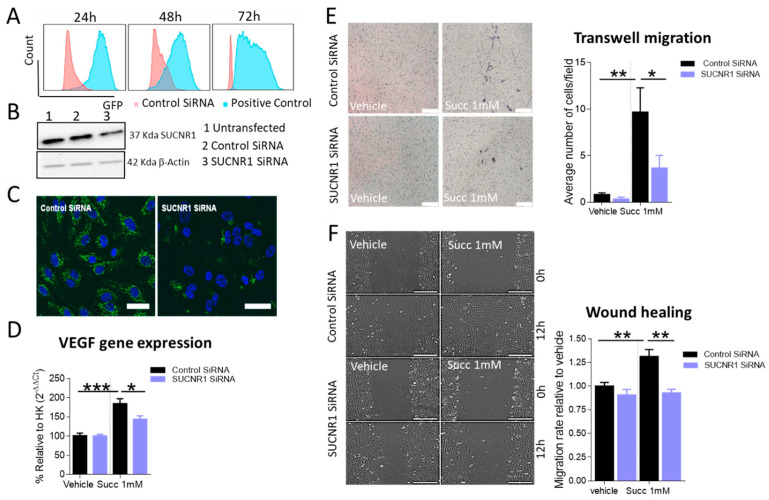
Knockdown of SUCNR1 suppresses the angiogenic phenotype of HUVECs. (**A**) Representative histogram of flow cytometric analysis of DY-547 labeled positive control SiRNA at 24, 48 and 72 h. (**B**) Representative Western blot of SUCNR1 in non-transfected, control or SUCNR1 SiRNA-transfected HUVECs at 72 h post transfection. (**C**) Representative image for immunofluorescence staining of SUCNR1 in HUVECs transfected with either control or SUCNR1 SiRNA for 72 h. DAPI was used for counterstaining (scale bar 10 µm, objective 20×). (**D**) RT-PCR of VEGF gene expression in control or SUCNR1 SiRNA-transfected HUVECs treated with either vehicle or succinate for 4 h. (**E**) Transwell migration assay of control or SUCNR1 SiRNA-transfected HUVECs in response to vehicle or succinate after 16 h; representative images are shown (scale bar 200 µm, objective 10×). (**F**) Scratch assay in control or SUCNR1 SiRNA-transfected HUVECs treated with vehicle or succinate for 12 h; representative images are shown (scale bar 500 µm, objective 10×). D, E and F were analyzed by two-way ANOVA followed by Tukey’s post hoc test, * *p* < 0.05, ** *p* < 0.01, *** *p* < 0.0001, data are shown as mean ±SEM (*n* = 3-5).

To investigate the specificity of our previous findings in HUVECs, we knocked down SUCNR1 using an SiRNA approach. We performed flow cytometric analysis of DY-547 labeled positive control SiRNA and our data showed the presence of SiRNA inside the cells at 24, 48 and 72 h (Figure 8A). We confirmed the knockdown efficacy at the protein level by Western blot and immunofluorescence staining of the receptor in control and SUCNR1 SiRNA-transfected cells at 72 h (Figure 8B,C). Functionally, knockdown of SUCNR1 significantly reduced VEGF gene expression in response to 1 mM succinate relative to control SiRNA-transfected cells at 4 h (Figure 8D). Moreover, the number of migrated cells after 16 h in response to succinate in a Transwell migration assay was significantly lower in SUCNR1 SiRNA-transfected HUVECs relative to control SiRNA (Figure 8E). In parallel, the wound healing capacity of SUCNR1 SiRNA-transfected HUVECs in response to succinate was significantly hampered relative to control transfected cells (Figure 8F). Collectively, knockdown of SUCNR1 prevented succinate induced VEGF gene expression, migration and wound closure in HUVECs. 

## 3. Discussion

Our study identifies, for the first time, an important axis comprised of succinate and its cognate receptor SUCNR1, as a potential driver of exuberant placental angiogenesis in GDM. Upregulation of succinate and its receptor in diabetic placental tissue lysates as well as a positive correlation of SUCNR1 and VEGF protein levels substantiated this hypothesis. At the cellular level, we confirmed the expression of SUCNR1 in human placental endothelial cells. Moreover, our functional experiments verified that succinate induces an angiogenic response both in HUVECs and FpECAs. Further, knockdown of SUCNR1 in HUVECs abolished this response implying that SUCNR1 is responsible for the angiogenic phenotype in response to succinate. 

In this study, we found that placentas from GDM pregnancies accumulated more succinate than normal placentas. Succinate accumulation under pathological conditions has been described in previous studies, where elevated succinate levels were detected in synovial joints of rheumatoid arthritis patients [28]. In addition, in models of diet-induced obesity, succinate in tissue and circulation was found to be elevated [29]. Increased succinate was also reported in certain tumors with succinate dehydrogenase mutations, the enzyme that metabolizes this intermediate [30]. Furthermore, higher plasma succinate concentrations in patients with T2D have been previously observed [19]. Besides, a metabolomics study using placental extracts measured higher succinate, among other metabolites, in preeclamptic placentas relative to controls [31]. However, mechanistic insights into the impact of dysregulated succinate levels on placental cellular functions in this pathology are still unavailable.

Moreover, our experiments unraveled increased SUCNR1 expression in GDM placenta tissue lysates. Likewise, upregulated SUCNR1 expression has been highlighted in other pathologies of immunological etiologies [32,33]. Whereas succinate–SUCNR1 interplay has been proposed as a molecular mechanism in diabetic neuroretinal angiopathy [20] as well as nephropathy [34], our data imply a role of this pathway also in placental angiogenesis through VEGF.

In this study, we focused on the placental endothelium as deciphering the underlying mechanisms of pregnancy pathologies with placental contribution lies, at least partly, in the endothelium which is in direct contact with fetal signals that regulate placental function [35]. It has been acknowledged that endothelial mitochondria play a role in sensing environmental signals rather than in energy production, since endothelial cells rely mainly on glycolysis for their energy needs [36]. 

Our data demonstrated expression of SUCNR1 in placental endothelial cells, which is in agreement with Toma et al. [34] and Zhang et al. [37], who described SUCNR1 expression in endothelial cells of the afferent arterioles of rabbit and mice kidney as well as human venous and arterial endothelial cells from the umbilical cord. Furthermore, we observed differential SUCNR1 expression between FpECAs and FpECVs. In fact, the placental endothelium is distinct in structure and function as veins carry oxygenated fetal blood from the mother, whereas deoxygenated blood from the fetus is carried through arteries [38], and dissimilarities between arterial and venous placental endothelial cells have been previously explored. Functionally, FpECAs showed more proliferative response to different VEGF isoforms in comparison to FpECVs [39]. Moreover, differences between these cells in response to GDM have been addressed using transcriptome and DNA methylation analyses showing that GDM induced cell-type specific alterations in actin organization and barrier function [40]. Further, microarray analysis highlighted transcriptional differences between HUVECs and human placental microvascular endothelial cells which was reflected in their responses to angiogenic stimuli as VEGF [41]. Interestingly, in normal pregnancies we found higher SUCNR1 expression in venous cells relative to arterial cells, which might seem surprising as placental arterial cells are more sensitive to angiogenic signals than venous cells [38]. A possible explanation is that the endothelial cells used in our experiments were isolated from term placentas and not earlier in pregnancy when angiogenesis mainly occurs. On the other hand, elevated SUCNR1 expression was observed in diabetic endothelial cells relative to cells from normal placentas although less prominently in venous cells as compared to arterial cells. We hypothesized that in a hyperglycemic environment, endothelial cells upregulated the receptor which led to sustained angiogenesis. This notion was supported by the observation that culture in high glucose induced SUCNR1 expression in HUVECs. 

Our flow cytometric data revealed weak expression of the receptor on the cell membrane, which is typical for most GPCRs, and that was corroborated with confocal microscopy. Interestingly, we observed that the receptor in non-stimulated cells was also expressed intracellularly and was coupled to the mitochondria. A similar observation has been made for another GPCR, the cannabinoid receptor CB(1) in mouse brain mitochondrial membranes of neurons, where it directly regulated energy production [42]. Classically, GPCRs were thought to be exclusively expressed at the cell surface where they transduce the signal to intracellular cascades. However, this view has been challenged as many GPCRs were found in different intracellular compartments, where they can get activated in situ [43,44,45]. It is of significance to note here that the form of succinate which we used in this study is non-permeable, so the possibility of ligand diffusion through the plasma membrane is minimal. However, whether this receptor fraction can be activated by de novo succinate synthesis in the mitochondria or by active transport of succinate from the extracellular space requires further investigation. 

Angiogenesis is a complex process that involves endothelial cell migration, proliferation and sprouting [46]. In our in vitro experiments, we addressed different endothelial cell angiogenesis correlates in response to succinate. For our study, we used succinate concentrations in the pathophysiological range (µM to mM). Succinate is measured in human systemic circulation in the low μM range [47]. However, it can accumulate up to mM concentration as in human gastric cancer tissues [21]. Pilot measurements of succinate concentrations in cord plasma from normal and GDM pregnancies in our laboratory showed a trend of succinate accumulation in the diabetic samples which was up to 100 µM in diabetic plasma (unpublished data). Our experiments in HUVECs confirmed the angiogenic potential of succinate. This could be further verified in FpECAs, where endothelial tube formation was already detected with lower succinate concentrations (30 µM). This observation supports the hypothesis of Hiden et al. [38], who attributed the sensitivity of placental arterial endothelial cells to angiogenic triggers to the requirement to expand fetal vasculature in proximity to the higher oxygen levels in the maternal blood, ensuring adequate supply to the fetus. Additionally, we demonstrated that both HUVECs and FpECAs responded to succinate by upregulating VEGF gene expression. The link between succinate–SUCNR1 signaling and VEGF has been addressed in earlier studies where succinate treatment through SUCNR1 induced VEGF production in retinal ganglion neurons [20], HUVECs [21] and rat aortic endothelial cells [22], signifying the importance of this receptor in angiogenesis. In fact, we found that the pro-angiogenic phenotype induced by succinate in HUVECs was suppressed when we knocked down SUCNR1, implicating that these responses are mediated through SUCNR1. 

Our data also showed that in EA.hy926 cells succinate induced ERK1/2 phosphorylation which are members of the MAPK/ERK pathway and regulate many cellular processes including proliferation, differentiation and cell responses to stress [48]. Similarly, activation of MAP kinases via SUCNR1 was described in macula dense cells in the kidney [49], retinal ganglion cell line (RGC-5) [50], human erythroleukaemia cell line (TF-1) [15] and cardiomyocytes [51].

In conclusion, our study sheds light on an extra-metabolic role of succinate as a signaling molecule in the placenta. Our results confirm the expression of SUCNR1 in placental endothelial cells and provide evidence that, in endothelial cells, succinate through its receptor triggers an angiogenic response which might be aggravated in GDM. Whether the succinate–SUCNR1 axis plays a role in other pregnancy pathologies such as preeclampsia is still to be addressed. Further investigation of alterations in placental metabolism and establishing clear causal relationships between tissue metabolism, succinate levels, SUCNR1 signaling and pregnancy pathologies is needed. We hypothesize that monitoring succinate levels in pregnancy might provide clinically useful cues towards pregnancy-related diseases, on the one hand, and targeting SUCNR1 in the placenta might open attractive novel therapeutic avenues, on the other hand.

## 4. Materials and Methods

All experiments requiring placental material from normal and GDM pregnancies were approved by the Ethical Committee of the Medical University of Graz (29-319 ex 16/17). All women who took part in the study provided written informed consent. All were non-smoking (self-reported). Normal pregnancy (PN) denoted a negative 75 g oral glucose tolerance test (OGTT) conducted at 25–28 weeks of gestation and had no other medical conditions or pregnancy complications. GDM was diagnosed following WHO/IADPSG measures. Women who had a positive OGTT were on diet control and classified as GDM A1 according to White’s Classification. Table 1 describes the clinical data of the pregnant women who participated in the study.

Chemicals were obtained from Sigma-Aldrich (St. Louis, MI, USA) unless specified otherwise. A list of all primary antibodies used in the study indicating their catalogue numbers is provided (Table S1).

### 4.1. Cell Culture

For functional assays, primary HUVECs were used due to limitation in access to sufficient isolations of primary fetoplacental endothelial cells (FpECs). HUVECs were purchased from Lonza (Basel, Switzerland) and grown in EGM-2 medium. For high glucose culture, HUVECs were cultivated in normal (5.5 mM) or high (20 mM) glucose in EGM-2 medium for 48 h. Primary FpEC isolations for addressing the receptor expression and validating the hypothesis were provided by the Department of Gynecology and Obstetrics at the Medical University of Graz. Placentas were collected directly after caesarean sections or normal deliveries, and primary FpECs were isolated as described by Lang et al. [39]. FpECs were cultured in PromoCell (Heidelberg, Germany) MV medium. Both HUVECs and FpECs were cultured at 21% O_2_, 5% CO_2_ and 37 °C in a 90% humidified incubator. Experiments were performed using cells between passages 3–7. EA.hy926 (ATCC, Manassas, VA, USA) at passages (13–17) were cultured as mentioned before in DMEM supplemented with 10% FBS, 1% *P*/S and 1% HAT media supplement (ThermoFisher Scientific, Waltham, MA, USA).

### 4.2. ISH and Immunofluorescence Microscopy of Tissue Sections 

Paraformaldehyde-fixed, paraffin embedded placenta sections were rehydrated. ISH was performed using RNAscope kit for SUCNR1 (purchased from ACD, Santa Ana, CA, USA) according to the manufacturer’s protocol as previously described [52]. The slides were blocked using 10% goat serum and 4% BSA in PBS + 0.3% Triton-X for 2 h. Sections were incubated overnight in primary antibody against VWF (Abcam, Cambride, UK). The 2nd antibody Alexa Fluor 488 goat anti-rabbit (Invitrogen, Waltham, MA, USA) was added after a washing step. Sections were mounted in DAPI-containing mounting medium (Vector Laboratories, Burlingame, CA, USA). Sections were analyzed with a confocal laser scanning microscope (Zeiss LSM 510 META, with a Plan-Neo 63x/1.4 Oil with DIC capability lens) and processed with ZEN software (Zeiss, Jena, Germany) and Fiji (version 1.51H).

### 4.3. Immunohistochemistry of Tissue Sections

Tissue sections were processed as described above. Primary antibody against VEGF (Abcam, Cambridge, UK) was used and the slides were counterstained with methyl green. Sections were visually examined with an Olympus BX41 microscope (Olympus Austria GmbH, Vienna, Austria) and an Olympus U Plan Apo ×20/0.25 lens. Analysis of immunohistochemistry images was performed with Fiji by splitting the acquired RGB Images into individual components and performing a flat field correction prior to automated thresholding. The results were then normalized on the total tissue area for each image.

### 4.4. Immunofluorescence Microscopy of FPECs/HUVECs 

Cells were seeded on chamber slides, grown until semi-confluence and processed as previously described [53]. In brief, cells were fixed in 3.7% paraformaldehyde for 10 min at room temperature with or without a subsequent permeabilization step with 0.1% Triton-X in PBS for 20 min. The cells were blocked in 10% 2nd antibody host serum with 4% BSA in PBS for 1 h at room temperature, followed by overnight incubation at 4 °C with SUCNR1 antibody (Novus Biologicals, Littleton, CO, USA). The cells were incubated with conjugated 2nd antibody (Invitrogen) for 2 h at room temperature after a washing step. For cell membrane localization, conjugated wheat germ agglutinin (WGA, ThermoFisher Scientific) or anti-VE-cadherin antibody (Santa Cruz Biotechnology, Dallas, TX, USA) was used. For subcellular receptor localization, we used CellLight Mitochondria-RFP (Invitrogen) to identify the mitochondria. The slides were mounted in DAPI-containing mounting medium and examined with confocal microscopy, z-stacks were acquired and images were processed with Fiji.

### 4.5. Flow Cytometry Surface and Intracellular Staining of SUCNR1 in HUVECs 

For surface staining, cells were blocked with human TruStain FcX (Biolegend, San Diego, CA, USA) for 10 min at 4 °C, while for the intracellular staining cells were fixed and permeabilized using fixation and permeabilization solution and perm/wash buffer (BD biosciences, Franklin Lakes, NJ, USA), respectively, before blocking. Cells were incubated with primary antibody against SUCNR1 (Novus Biologicals, Littleton, CO, USA) for 45 min at 4 °C. Afterwards, the cells were incubated with conjugated 2nd antibody (Invitrogen, Waltham, MA, USA) for 45 min at 4 °C. The cells were suspended in PBS and the positive cells were counted using BD FACSCanto II. Analysis was performed using FlowJo software (version 10.7.1).

### 4.6. Western Blot 

Tissue/cells were lysed in RIPA extraction buffer (ThermoFisher Scientific) supplemented with 1X Protease and Phosphatase Inhibitor Cocktail (ThermoFisher Scientific). Protein concentration was measured using Pierce BCA-kit (ThermoFisher Scientific) and protein samples were separated by SDS-PAGE on a 4–20% TRIS-glycine gradient gel (ThermoFisher Scientific). Afterwards, the proteins were blotted onto a polyvinylidene fluoride membrane using iBlot system (Invitrogen). Target proteins were detected using specific antibodies and visualized with respective horseradish peroxidase (HRP) conjugated antibodies (Cell signaling, Danvers, MA, USA) and Clarity Western ECL substrate (Biorad, Hercules, CA, USA). Chemiluminescence was recorded by ChemiDoc Touch Imaging system (Biorad). After stripping the membranes using Restore Western Blot Stripping Buffer (ThermoFisher Scientific) for 20 min, membranes were reprobed with β-Actin antibody (Cell signaling) and further processed as described above. Densitometric analysis of protein bands was performed using Imagelab Software (version 5.2). Western blot images were cropped for easier presentation. Originals are visualized in the supplementary material.

### 4.7. Apoptosis Assay 

HUVECs were incubated with either vehicle or succinate for different time points. Annexin V (Biolegend) and propidium iodide (ThermoFisher Scientific) co-staining was performed and analysis was done using BD FACSCanto II and FlowJo software.

### 4.8. JC-1 Staining 

HUVECs were treated with vehicle or succinate for different time points, stained with JC-1 dye (ThermoFisher Scientific) for 10 min in the dark and analyzed using BD FACSCanto II and FlowJo software. Carbonyl cyanide-4-(trifluoromethoxy) phenylhydrazone (FCCP, Abcam) was used as a positive control. The ratio of JC monomers to aggregates, indicative of the mitochondrial membrane potential, was calculated.

### 4.9. Flow Cytometric Staining of p-ERK1/2 

EA.hy926 cells were serum starved for 16 h before treatment with 10 mM succinate for indicated time points. The cells were detached with Accutase on ice and then fixed with phosphoflow fix buffer for 10 min at 37 °C and permeabilized with phosphoflow perm buffer (both from BD Biosciences) for 30 min on ice. The cells were blocked with 0.5% BSA in PBS and antibody against pERK (Cell signaling) was used for 30 min on ice. Conjugated 2nd antibody was used for 30 min on ice after a washing step. Thirty thousand cells per condition were measured by flow cytometry (FACSCanto II). Data were calculated as fold increase in fluorescence intensity with respect to vehicle.

### 4.10. EdU Proliferation Assay 

Cell proliferation was evaluated using EdU proliferation kit (ThermoFisher Scientific) according to the manufacturer’s instructions. The number of EdU positive cells relative to the total number of cells was counted in 5 different fields by an independent blinded researcher.

### 4.11. Real-Time Polymerase Chain Reaction (RT-PCR) 

For relative quantification of mRNA, real-time PCR was performed using (CDX Connect Real-Time PCR detection system with CFX Manager Software 3.1; Biorad). RNA isolation was done using Trizol (ThermoFisher Scientific) and DNA was removed with Ambion DNA removal kit (ThermoFisher Scientific). RNA was reverse transcribed using the iScript cDNA synthesis kit (Biorad). Real-time PCR was performed using SsoAdvanced universal SYBR Green Supermix and PrimePCR SYBR Green Assay primers (Biorad) for human VEGF (Unique Assay ID: qHsaCED0006937) and GAPDH (Unique assay ID: qHsaCED0038674) according to the manufacturer’s instructions.

### 4.12. Enzyme-Linked Immunosorbent Assay (ELISA) 

Conditioned media from HUVECs (10^5^ cells) treated with vehicle or succinate for 24 h were collected. VEGF was measured using ELISA Kit (R&D Systems, Minneapolis, MN, USA) according to the manufacturer’s instructions. Each sample was tested in triplicate.

### 4.13. Transwell Migration Assay 

HUVECs were seeded in the upper chambers of Transwell plates and the bottom chambers were filled with media containing vehicle or succinate. After 16 h of culture, the non-migrated cells were removed, and the migrated cells were fixed, permeabilized and stained with trypan blue. Images from 5 different fields were taken and the average cell number per field was calculated.

### 4.14. Scratch Wound Healing Assay 

HUVECs were seeded in gelatin-coated 12-well plates and allowed to grow to full confluence. The cell monolayer was wounded with 200 µL pipette tips and cultured in media with or without succinate. Time-lapse imaging was used to assess the migration of the cells for 12 h at 37 °C in a 95%:5% (*v/v*) mixture of air and CO_2_ (Nikon Ti-2 microscope equipped with an Andor Zyla 4.2 sCMOS camera). Images were later analyzed for the area taken up by the cells; these results were plotted and a function was fitted. The inclination of the functions was used as a readout for migration speed as the inclination is independent of the offset (starting gap size).

### 4.15. Succinate Measurement 

Succinate was quantified in tissue lysates (10 mg) using succinate colorimetric assay kit (Abcam) according to the manufacturer’s instructions. 

### 4.16. Tube Formation Fibrin Angiogenesis Assay 

Tube formation assay was performed as described [54,55]. Briefly, FpECAs were seeded onto a fibrin matrix composed of 0.4% fibrinogen and 0.03 U/mL Thrombin IIa (CoaChrom Diagnostica, Maria Enzersdorf, Austria). After overnight incubation, medium was replaced by media supplemented with succinate either alone or in combination with the positive control bVT, a combination of TNFα (10 ng/mL, ReliaTech, Wolfenbüttel, Germany), FGF2 (10 ng/mL, ReliaTech) and VEGF (25 ng/mL; ReliaTech). After 48 h, the assay was terminated using 3.7% formaldehyde for 2 h which was then replaced with PBS. Z-stacks were acquired with a step size of 10µm using a Nikon Ti-2 microscope. Images were analyzed using Fiji and tube length per mm^2^ was calculated.

### 4.17. Spheroid Sprouting Angiogenesis Assay 

HUVECs were suspended in 0.3% methylcellulose solution and spheroids in hanging drops were generated. A collagen-spheroid suspension from (0.7% methylcellulose), NaHCO_3_ (15.6 mg/mL), Type 1 collagen (2 mg/mL) and NaOH (1 M) was pipetted in a 24-well plate and incubated at 37 °C, 21% O_2_ for 2 h to allow collagen polymerization. Stimulation media containing vehicle or succinate was added and bVT was used as positive control. Spheroids were stimulated for 16 h and the assay was terminated using 3.7% formaldehyde for 2 h at room temperature and then replaced with PBS. Automated z-stack on Nikon HCS were generated using the 10× objective. The numbers of sprouts as well as total sprout length were calculated with Fiji.

### 4.18. Small Interfering (SiRNA) Transfection 

SUCNR1 knockdown was carried out using on-target plus human SUCNR1 SiRNA (mixture of 4 different siRNA, Dharmacon, Lafayette, CO, USA) and Dharmafect 4 transfection reagent according to the manufacturer’s instructions. siGLO Lamin A/C SiRNA DY-547 labeled and on-target plus non-targeting pool were used as positive and negative controls, respectively. 50 nM of SiRNA was used for 50,000 cells and the efficiency of transfection was determined by analyzing SUCNR1 protein using Western blot as well as immunofluorescence.

### 4.19. Statistical Analysis 

GraphPad Prism software 6.0 (La Jolla, CA, USA) was used. Unpaired *t*-test was used to compare the means of 2 independent groups, while a paired *t*-test was used to compare the means from the same group under 2 different experimental conditions. For more than 2 groups, one-way or two-way ANOVA was used based on the number of variables followed by Tukey or Dunnett multi-comparison test. 

## Figures and Tables

**Table 1:** Clinical features of normal and GDM pregnancies. Samples were matched based on gestational age, body mass index (BMI) before pregnancy as well as the absence of other pregnancy complications. Gestational age and BMI are shown as mean ± SD. ns denotes not statistically significant.

	PN (*n* = 9)	GDM (*n* = 7)	*p* Value
Gestational age (Weeks)	38.1 ± 0.9	38.8 ± 0.8	ns
BMI before pregnancy	22.9 ± 2.8	26.8 ± 4.8	ns
Other pregnancy complications	None	None	

## Data Availability

The data generated in the study are presented in the article and Supplementary Material. Requests of different nature can be directed to the corresponding author on a reasonable basis.

## Supplementary Materials

The following are available online at https://www.mdpi.com/article/10.3390/ijms222112048/s1, Table S1: List of primary antibodies used in the study and their cat numbers, Figure S1: HUVECs express similar SUCNR1 levels as FpECVs but comparatively lower levels than HEK293 cells and human monocytes derived macrophages, Figure S2: Succinate fails to induce AKT phosphorylation in EA.hy926 cells.

## References

[1] Burton G.J., Charnock-Jones D.S., Jauniaux E. (2009). Regulation of vascular growth and function in the human placenta. Reproduction.

[2] Jirkovská M., Janáček J., Kaláb J., Kubínová L. (2008). Three-dimensional Arrangement of the Capillary Bed and Its Relationship to Microrheology in the Terminal Villi of Normal Term Placenta. Placenta.

[3] Demir R., Kayisli U.A., Seval Y., Celik-Ozenci C., Korgun E.T., Demir-Weusten A.Y., Huppertz B. (2004). Sequential expression of VEGF and its receptors in human placental villi during very early pregnancy: Differences between placental vasculogenesis and angiogenesis. Placenta.

[4] Aye I.L.M.H., Aiken C.E., Charnock-Jones D.S., Smith G.C.S. (2020). Placental energy metabolism in health and disease—Significance of development and implications for preeclampsia. Am. J. Obstet. Gynecol..

[5] McElwain C.J., Tuboly E., McCarthy F.P., McCarthy C.M. (2020). Mechanisms of Endothelial Dysfunction in Pre-eclampsia and Gestational Diabetes Mellitus: Windows Into Future Cardiometabolic Health?. Front. Endocrinol..

[6] Shou C., Wei Y.-M., Wang C., Yang H.-X. (2019). Updates in Long-term Maternal and Fetal Adverse Effects of Gestational Diabetes Mellitus. Matern. Med..

[7] Huynh J., Dawson D., Roberts D., Bentley-Lewis R. (2015). A systematic review of placental pathology in maternal diabetes mellitus. Placenta.

[8] Cvitic S., Desoye G., Hiden U. (2014). Glucose, Insulin, and Oxygen Interplay in Placental Hypervascularisation in Diabetes Mellitus. Biomed. Res. Int..

[9] Muralimanoharan S., Maloyan A., Myatt L. (2016). Mitochondrial function and glucose metabolism in the placenta with gestational diabetes mellitus: Role of miR-143. Clin. Sci..

[10] Abbade J., Klemetti M.M., Farrell A., Ermini L., Gillmore T., Sallais J., Tagliaferro A., Post M., Caniggia I. (2020). Increased placental mitochondrial fusion in gestational diabetes mellitus: An adaptive mechanism to optimize feto-placental metabolic homeostasis?. BMJ Open Diabetes Res. Care.

[11] Krebs H.A., Johnson W.A. (1937). The role of citric acid in intermediate metabolism in animal tissues. Enzymologia.

[12] Hoyer S., Krier C. (1986). Ischemia and the aging brain. Studies on glucose and energy metabolism in rat cerebral cortex. Neurobiol. Aging.

[13] He W., Miao F.J.P., Lin D.C.H., Schwandner R.T., Wang Z., Gao J., Chen J.L., Tlan H., Ling L. (2004). Citric acid cycle intermediates as ligands for orphan G-protein-coupled receptors. Nature.

[14] Correa P.R.A.V., Kruglov E.A., Thompson M., Leite M.F., Dranoff J.A., Nathanson M.H. (2007). Succinate is a paracrine signal for liver damage. J. Hepatol..

[15] Hakak Y., Lehmann-Bruinsma K., Phillips S., Le T., Liaw C., Connolly D.T., Behan D.P. (2009). The role of the GPR91 ligand succinate in hematopoiesis. J. Leukoc. Biol..

[16] An Y.A., Chen S., Deng Y., Wang Z.V., Funcke J.B., Shah M., Shan B., Gordillo R., Yoshino J., Klein S. (2021). The mitochondrial dicarboxylate carrier prevents hepatic lipotoxicity by inhibiting white adipocyte lipolysis. J. Hepatol..

[17] Aguiar C.J., Andrade V.L., Gomes E.R.M., Alves M.N.M., Ladeira M.S., Pinheiro A.C.N., Gomes D.A., Almeida A.P., Goes A.M., Resende R.R. (2010). Succinate modulates Ca2+ transient and cardiomyocyte viability through PKA-dependent pathway. Cell Calcium.

[18] Peti-Peterdi J. (2010). High glucose and renin release: The role of succinate and GPR91. Kidney Int..

[19] van Diepen J.A., Robben J.H., Hooiveld G.J., Carmone C., Alsady M., Boutens L., Bekkenkamp-Grovenstein M., Hijmans A., Engelke U.F.H., Wevers R.A. (2017). SUCNR1-mediated chemotaxis of macrophages aggravates obesity-induced inflammation and diabetes. Diabetologia.

[20] Sapieha P., Sirinyan M., Hamel D., Zaniolo K., Joyal J.S., Cho J.H., Honoré J.C., Kermorvant-Duchemin E., Varma D.R., Tremblay S. (2008). The succinate receptor GPR91 in neurons has a major role in retinal angiogenesis. Nat. Med..

[21] Mu X., Zhao T., Xu C., Shi W., Geng B., Shen J., Zhang C., Pan J., Yang J., Hu S. (2017). Oncometabolite succinate promotes angiogenesis by upregulating VEGF expression through GPR91-mediated STAT3 and ERK activation. Oncotarget.

[22] Li Y., Liu Y., Wang C., Xia W.R., Zheng J.Y., Yang J., Liu B., Liu J.Q., Liu L.F. (2018). Succinate induces synovial angiogenesis in rheumatoid arthritis through metabolic remodeling and HIF-1α/VEGF axis. Free Radic. Biol. Med..

[23] Gilissen J., Jouret F., Pirotte B., Hanson J. (2016). Insight into SUCNR1 (GPR91) structure and function. Pharmacol. Ther..

[24] Betz C., Lenard A., Belting H.G., Affolter M. (2016). Cell behaviors and dynamics during angiogenesis. Development.

[25] Geubelle P., Gilissen J., Dilly S., Poma L., Dupuis N., Laschet C., Abboud D., Inoue A., Jouret F., Pirotte B. (2017). Identification and pharmacological characterization of succinate receptor agonists. Br. J. Pharmacol..

[26] Ferrara N., Gerber H.P., LeCouter J. (2003). The biology of VEGF and its receptors. Nat. Med..

[27] Icli B., Feinberg M.W. (2016). Plasticity of Arterial and Venous Endothelial Cell Identity: Some Nerve!. Circ. Res..

[28] Kim S., Hwang J., Xuan J., Jung Y.H., Cha H.S., Kim K.H. (2014). Global metabolite profiling of synovial fluid for the specific diagnosis of rheumatoid arthritis from other inflammatory arthritis. PLoS ONE.

[29] Sadagopan N., Li W., Roberds S.L., Major T., Preston G.M., Yu Y., Tones M.A. (2007). Circulating Succinate is Elevated in Rodent Models of Hypertension and Metabolic Disease. Am. J. Hypertens..

[30] Zhao T., Mu X., You Q. (2017). Succinate: An initiator in tumorigenesis and progression. Oncotarget.

[31] Dunn W.B., Brown M., Worton S.A., Davies K., Jones R.L., Kell D.B., Heazell A.E.P. (2012). The metabolome of human placental tissue: Investigation of first trimester tissue and changes related to preeclampsia in late pregnancy. Metabolomics.

[32] Macias-Ceja D.C., Ortiz-Masiá D., Salvador P., Gisbert-Ferrándiz L., Hernández C., Hausmann M., Rogler G., Esplugues J.V., Hinojosa J., Alós R. (2019). Succinate receptor mediates intestinal inflammation and fibrosis. Mucosal Immunol..

[33] Zhang J., Zhang Q., Yang Y., Wang Q. (2020). Association Between Succinate Receptor SUCNR1 Expression and Immune Infiltrates in Ovarian Cancer. Front. Mol. Biosci..

[34] Toma I., Kang J.J., Sipos A., Vargas S., Bansal E., Hanner F., Meer E., Peti-Peterdi J. (2008). Succinate receptor GPR91 provides a direct link between high glucose levels and renin release in murine and rabbit kidney. J. Clin. Investig..

[35] Elad D., Levkovitz R., Jaffa A.J., Desoye G., Hod M. (2014). Have We Neglected the Role of Fetal Endothelium in Transplacental Transport?. Traffic.

[36] Caja S., Enríquez J.A. (2017). Mitochondria in endothelial cells: Sensors and integrators of environmental cues. Redox Biol..

[37] Zhang H., Zheng J., Lin J., Chen J., Yu Z., Chen C., Liu T. (2018). miR-758 mediates oxLDL-dependent vascular endothelial cell damage by suppressing the succinate receptor SUCNR1. Gene.

[38] Hiden U., Lang I., Ghaffari-Tabrizi N., Gauster M., Lang U., Desoye G. (2009). Insulin Action on the Human Placental Endothelium in Normal and Diabetic Pregnancy. Curr. Vasc. Pharmacol..

[39] Lang I., Schweizer A., Hiden U., Ghaffari-Tabrizi N., Hagendorfer G., Bilban M., Pabst M.A., Korgun E.T., Dohr G., Desoye G. (2008). Human fetal placental endothelial cells have a mature arterial and a juvenile venous phenotype with adipogenic and osteogenic differentiation potential. Differentiation.

[40] Cvitic S., Novakovic B., Gordon L., Ulz C.M., Mühlberger M., Diaz-Perez F.I., Joo J.E., Svendova V., Schimek M.G., Trajanoski S. (2018). Human fetoplacental arterial and venous endothelial cells are differentially programmed by gestational diabetes mellitus, resulting in cell-specific barrier function changes. Diabetologia.

[41] Huang X., Jia L., Qian Z., Jia Y., Chen X., Xu X., Chang X., Liu M., Wang K. (2018). Diversity in human placental microvascular endothelial cells and macrovascular endothelial cells. Cytokine.

[42] Bénard G., Massa F., Puente N., Lourenço J., Bellocchio L., Soria-Gómez E., Matias I., Delamarre A., Metna-Laurent M., Cannich A. (2012). Mitochondrial CB 1 receptors regulate neuronal energy metabolism. Nat. Neurosci..

[43] Jong Y.J.I., Harmon S.K., O’Malley K.L. (2018). GPCR signalling from within the cell. Br. J. Pharmacol..

[44] Campden R., Audet N., Hébert T.E. (2015). Nuclear G protein signaling: New tricks for old dogs. J. Cardiovasc. Pharmacol..

[45] Jong Y.J.I., Sergin I., Purgert C.A., O’Malley K.L. (2014). Location-dependent signaling of the group 1 metabotropic glutamate receptor mGlu5. Mol. Pharmacol..

[46] Koch A.E., Distler O. (2007). Vasculopathy and disordered angiogenesis in selected rheumatic diseases: Rheumatoid arthritis and systemic sclerosis. Arthritis Res. Ther..

[47] Kushnir M.M., Komaromy-Hiller G., Shushan B., Urry F.M., Roberts W.L. (2001). Analysis of dicarboxylic acids by tandem mass spectrometry. High-throughput quantitative measurement of methylmalonic acid in serum, plasma, and urine. Clin. Chem..

[48] Guo Y., Pan W., Liu S., Shen Z., Xu Y., Hu L. (2020). ERK/MAPK signalling pathway and tumorigenesis (Review). Exp. Ther. Med..

[49] Vargas S.L., Toma I., Jung J.K., Meer E.J., Peti-Peterdi J. (2009). Activation of the succinate receptor GPR91 in macula densa cells causes renin release. J. Am. Soc. Nephrol..

[50] Hu J., Li T., Du S., Chen Y., Wang S., Xiong F., Wu Q. (2015). The MAPK signaling pathway mediates the GPR91-dependent release of VEGF from RGC-5 cells. Int. J. Mol. Med..

[51] Aguiar C.J., Rocha-Franco J.A., Sousa P.A., Santos A.K., Ladeira M., Rocha-Resende C., Ladeira L.O., Resende R.R., Botoni F.A., Melo M.B. (2014). Succinate causes pathological cardiomyocyte hypertrophy through GPR91 activation. Cell Commun. Signal..

[52] Bärnthaler T., Theiler A., Zabini D., Trautmann S., Stacher-Priehse E., Lanz I., Klepetko W., Sinn K., Flick H., Scheidl S. (2020). Inhibiting eicosanoid degradation exerts antifibrotic effects in a pulmonary fibrosis mouse model and human tissue. J. Allergy Clin. Immunol..

[53] Bärnthaler T., Maric J., Platzer W., Konya V., Theiler A., Hasenöhrl C., Gottschalk B., Trautmann S., Schreiber Y., Graier W.F. (2017). The Role of PGE2 in Alveolar Epithelial and Lung Microvascular Endothelial Crosstalk. Sci. Rep..

[54] Leopold B., Strutz J., Weiß E., Gindlhuber J., Birner-Gruenberger R., Hackl H., Appel H.M., Cvitic S., Hiden U. (2019). Outgrowth, proliferation, viability, angiogenesis and phenotype of primary human endothelial cells in different purchasable endothelial culture media: Feed wisely. Histochem. Cell Biol..

[55] Koolwijk P., Van Erck M.G.M., De Vree W.J.A., Vermeer M.A., Weich H.A., Hanemaaijer R., Van Hinsbergh V.W.M. (1996). Cooperative effect of TNFα, bFGF, and VEGF on the formation of tubular structures of human microvascular endothelial cells in a fibrin matrix. Role of urokinase activity. J. Cell Biol..

