# Supplementary material for: SUCNR1 Is Expressed in Human Placenta and Mediates Angiogenesis: Significance in Gestational Diabetes
Source: Int J Mol Sci. 2021 Nov 7;22(21):12048. doi: 10.3390/ijms222112048 (PMC8585094; doi:10.3390/ijms222112048)
Supplement: Supplementary file 1 [file ijms-22-12048-s001.zip › ijms-1434366-supplementary.pdf]

SUCNR1 is expressed in human placenta and mediates angiogenesis: Significance in gestational diabetes.

Supplementary material

Table S1. List of primary antibodies used in the study and their catalogue numbers.

Antibody	Company	Cat #
SUCNR1 (WB)	Novus Biologicals	NBP2-82350
SUCNR1 (IF, Flow)	Novus Biologicals	NLS3476
VEGF	Abcam	ab51745
VE-Cad	Santa Cruz	sc-9989
VWF	Abcam	ab6994
β-Actin	Cell Signaling	mAB3700
P-ERK	Cell signaling	4370S
P-AKT	Cell signaling	4060S

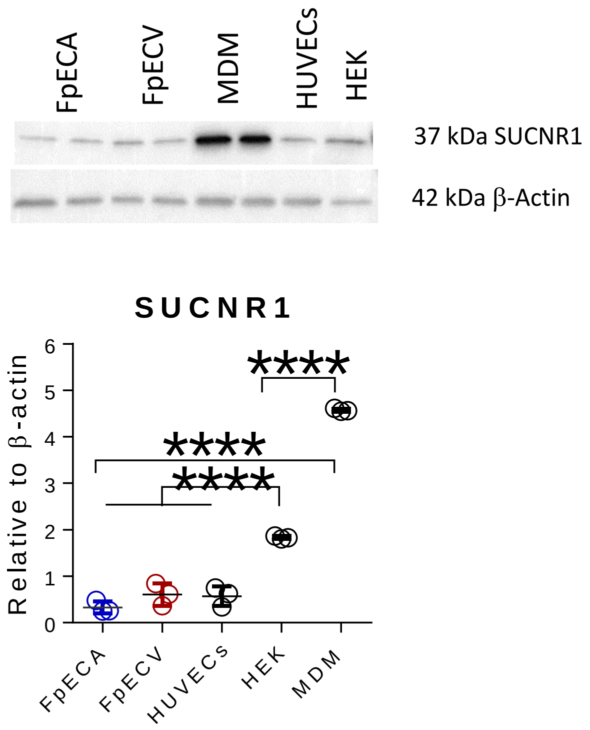

Figure S1. HUVECs express similar SUCNR1 levels as FpECVs but comparatively lower levels than HEK293 cells and human monocytes derived macrophages. Western blot of SUCNR1 and densitometric analysis in different cell models expressing the receptor analyzed by one-way ANOVA followed Tukey's post-hoc test, **** $p<0.0001$, data are shown as mean ± SD (n=3). HEK cells (passage 16, originally from Jennifer Whistler lab (UCSF)) and monocyte derived macrophages (isolated and differentiated from healthy donors) used for Western blot were accessible from another running study.

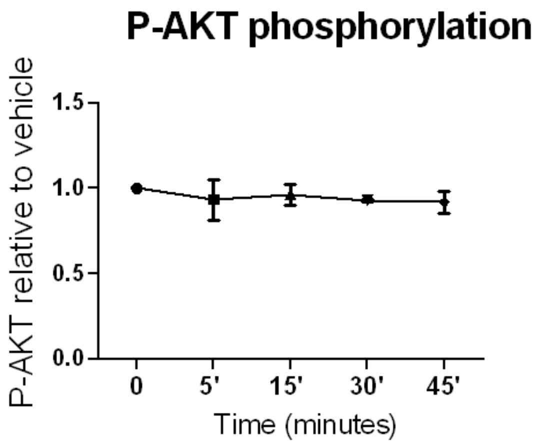

Figure S2. Succinate fails to induce AKT phosphorylation in EA.hy926 cells. Data are expressed as fold change in fluorescence intensity relative to vehicle. Data are shown as mean ± SEM (n=3).
